# Strategies for *in vitro* engineering of the translation machinery

**DOI:** 10.1093/nar/gkz1011

**Published:** 2019-11-28

**Authors:** Michael J Hammerling, Antje Krüger, Michael C Jewett

**Affiliations:** Department of Chemical and Biological Engineering, Center for Synthetic Biology, Northwestern University, 2145 Sheridan Road, Evanston, IL 60208, USA

## Abstract

Engineering the process of molecular translation, or protein biosynthesis, has emerged as a major opportunity in synthetic and chemical biology to generate novel biological insights and enable new applications (e.g. designer protein therapeutics). Here, we review methods for engineering the process of translation *in vitro*. We discuss the advantages and drawbacks of the two major strategies—purified and extract-based systems—and how they may be used to manipulate and study translation. Techniques to engineer each component of the translation machinery are covered in turn, including transfer RNAs, translation factors, and the ribosome. Finally, future directions and enabling technological advances for the field are discussed.

## INTRODUCTION

**Figure 1.**
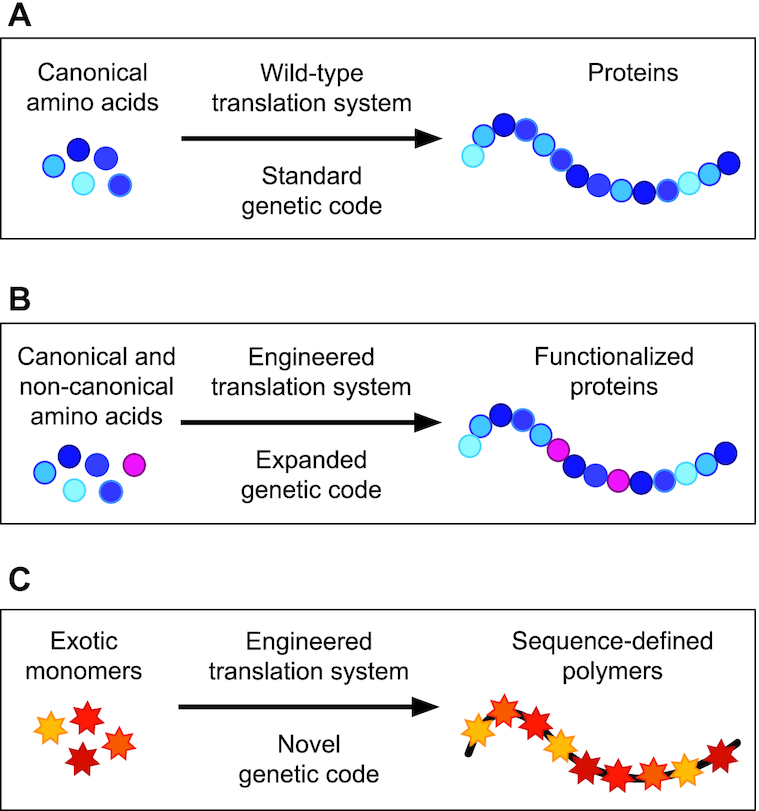
Conceptual goals for engineering templated polymer production by the ribosome. (**A**) The standard genetic code enables the polymerization of canonical amino acids with a diversity of 20 proteinogenic side chains (blue shaded circles). Despite enabling the evolution of life and having been harnessed for societal needs (e.g. recombinant protein production of protein therapeutics like insulin), protein biosynthesis in nature uses limited sets of protein monomers, which results in limited sets of biopolymers (i.e. proteins). (**B**) First-generation genetic code expansion facilitates the incorporation of L-α-amino acids with a vast array of chemical side chains into proteins (pink circles). Site-specific incorporation of up to 40 instances of a single ncAA in a single polypeptide chain has been reported (10). (**C**) Next-generation genetic code expansion involves the incorporation of monomers with both non-canonical side chains and backbones (multi-colored stars). Engineering of all aspects of the translation apparatus will be required to generate systems capable of efficiently carrying out polymerization of these exotic new molecules.

The translation machinery—the ribosome and associated factors necessary for protein biosynthesis—polymerizes l-α-amino acid building blocks into proteins according to instructions presented in messenger RNA (mRNA) and defined by the genetic code (Figure 1A). Given the redundancy of the genetic code (i.e. 64 codons encode only 20 amino acids), there has long been interest in understanding how one might re-engineer the genetic code to incorporate monomers with novel side chain chemistries or backbones (1–5). Key among efforts to reprogram the genetic code are pioneering studies that have shown the flexibility of the translation system to incorporate non-canonical amino acids (ncAA) into biopolymers using genetic code expansion approaches (6–10) (Figure 1B). Recently, substantial advances have been made in the considerably more challenging task of incorporating monomers with novel backbones, including *N*-methyl- (7,11–14), α-hydroxy acid (15,16), d-α- (17,18) and β-amino acids (19,20), as well as polypeptoids (21) and even foldamers (22). Such monomers allow modulation of not just side chain chemistry but polymer folding and stability properties as well (Figure 1C). Considering these possibilities, expanding the genetic code for the synthesis of novel sequence-defined polymers has emerged as a major opportunity in synthetic and chemical biology (23).

Engineering the translation machinery is a complex and formidable challenge for many reasons. Here, we highlight three. First, translation involves the interplay of dozens of individual proteins and RNAs, and due to the challenge of optimizing all these components simultaneously, most studies have focused on altering only one or two components at a time (24–26). Second, protein translation with non-canonical monomers often suffers from poor efficiencies and low yields of full-length product, especially when incorporating multiple, distinct ncAAs (27). Third, biological constraints limit the scope of permissible engineering possibilities to expand ncAA diversity. An especially challenging constraint is the limited mutability of the ribosome, since ribosome function must be preserved to maintain cell viability. This restricts the mutations that can be made to the ribosome, thus excluding many ribosomal RNA (rRNA) genotypes that may enable new ribosome function, but are incompatible with the viability of living cells (28).

To address the complex issues above, new tools are needed to derive general paradigms for engineering translation systems. Though most efforts to engineer the translation system have been pursued *in vivo* (i.e. in living cells), *in vitro* (i.e. cell-free) approaches have several key benefits. First, they do not suffer from cell-viability constraints, facilitating the use of toxic genotypes (e.g. orthogonal translation system components required to expand the genetic code and incorporate ncAAs) and non-physiological reaction conditions (29). Second, cell-free systems allow precise control of reaction conditions and permit the addition and removal of individual components to study their effects on translation (30). Third, they enable rapid, automation-assisted assembly of reactions from individual components for efficient system optimization (31). Taken together, these features provide a freedom of design and control that make cell-free systems an attractive complement to cellular approaches for studying and engineering translation.

This review aims to provide an overview of recent advances for engineering the translation machinery *in vitro*. We begin by covering the two general platforms for *in vitro* protein translation: the PURE system (i.e. protein synthesis using purified recombinant elements) and extract-based systems. We then examine strategies for engineering each non-ribosomal component of the translational system, including transfer RNAs (tRNAs) and translation factors. We next cover strategies for the reconstitution and *in vitro* synthesis of the ribosome, which set the stage for engineering the central catalyst of translation. Finally, we review recent technological advances that will impact *in vitro* translation engineering and discuss the future outlook of the field. Overall, this review is intended to provide a focused perspective on the past, current, and future challenges of *in vitro* translation engineering for those researchers wishing to learn about and influence this rapidly developing field.

## *IN VITRO* PROTEIN TRANSLATION PLATFORMS

*In vitro* translation systems facilitate the biosynthesis of recombinant proteins without using intact cells. In recent years, improvements in such systems have enabled accurate and efficient incorporation of ncAAs into proteins for genetic code expansion. Two main platforms have been developed: the PURE system and the extract-based system.

### The PURE translation system

**Figure 2.**
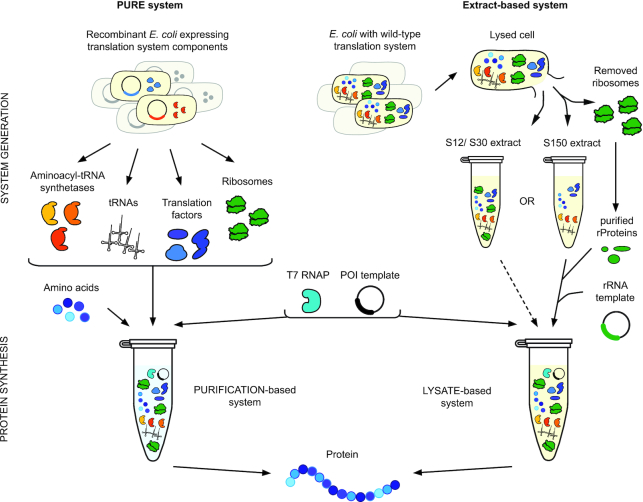
*In vitro* protein synthesis systems facilitate translation system engineering. Two strategies exist for enabling protein translation *in vitro*: the PURE system and extract-based systems. In the PURE system (left), each unique component of the translation apparatus is individually purified from cells, including the aminoacyl-tRNA synthetases, tRNAs, translation factors, and ribosomes. In order to reconstitute a functional translation system, these components are then recombined together with amino acids, energy substrates, cofactors, salts, a template for a protein of interest (POI), and T7 RNA polymerase (RNAP) to generate mRNA template. Importantly, this methodology enables precise optimization of component concentrations and the ability to leave out certain components and replace them with modified components to modulate translation apparatus function. In contrast, extract-based systems (right) entail a simpler protocol for the preparation of a crude cellular extract containing all the necessary components.

In the PURE system, all the translation factors, tRNAs, components for mRNA template generation, and ribosomes are individually purified from cells and assembled *in vitro* to create a translationally competent environment (30) (Figure 2, left). This strategy enables the user to define the concentrations and genotypes of all components in the translation reaction. The exquisite control afforded by the PURE system has spawned a variety of synthetic biology platforms which leverage this capability (32). For example, Suga *et al.*’s pioneering efforts have used the flexibility for genetic code reprogramming available in the PURE system for highly efficient sense and non-sense suppression in incorporating ncAAs into peptidomimetic drugs (21,33–35). Additionally, Forster *et al.* showed the ability to program peptidomimetics by translating genetic codes designed *de novo* (36).

However, this approach entails several challenges that have been addressed to varying degrees. First, determining the ideal concentration of each translation component is a difficult optimization problem. A systematic analysis of interactions between the concentrations of 69 translation components enabled optimization of the concentration of those components in the PURE system (37). Subsequent improvements resulted in protein yields of 4.4 g/l of β-galactosidase in a semi-continuous reaction (38) or a 5-fold improvement in luciferase production from translation factor optimization and a further ∼2-fold improvement by replenishing six small molecule substrates (39,40). A second challenge is the high relative cost of the PURE system compared with extract-based systems. Estimates suggest that PURE is ∼2 orders of magnitude more expensive per gram of protein produced than extract-based approaches (41). Recently published techniques to simplify the process of generating PURE system components can substantially reduce costs and labor (42–44), but since all these techniques use one-pot purifications, they also necessarily entail some loss in control and modularity over translation components.

### Extract-based systems

The history of extract-based *in vitro* translation systems is rooted in the origins of molecular biology, as such systems were used to elucidate the genetic code (45,46). Recently, extract-based protein synthesis methods have enjoyed a resurgence in interest driven by advances in system capabilities such as high-level protein expression (> g/l) for prototyping and characterizing biological systems (47–52), on-demand biomanufacturing (53–57), glycoprotein synthesis (58,59), molecular diagnostics (60–64) and education (65–68), among others (reviewed in (69,70)).

While a variety of cell-free reaction preparation methods exist, each generally involves lysis and the extraction of the crude intracellular milieu, supplementation with enhancing components such as cofactors and an energy source, and protein synthesis from a DNA template (Figure 2, right). As a platform for engineering translation, the primary advantage of extract-based methodologies is the ability to obtain the entire complement of translation machinery components with a simple extraction to remove cell wall debris and chromosomal DNA. This method also retains ancillary components that aid functional protein synthesis, such as recycling enzymes, metabolic enzymes, chaperones, and foldases. These components may account for the ability of extract-based systems to produce more protein per ribosome than the PURE approach.

While crude extract-based systems offer simplicity of preparation, the difficulty of completely defining the translational environment is a drawback. Exerting greater control over extract-based systems entails more involved extract processing, including selective depletion of components of the translation machinery. For example, depletion of tRNAs via degradation (71,72) or DNA-hybridization chromatography (73), or inactivation of tRNAs via sequestration using synthetic oligonucleotides (74) can be used to reassign the meaning of sense codons in extracts. Similarly, removal of native ribosomes via ultracentrifugation (i.e. 150 000 × *g*) is the basis for a platform that can build ribosomes *in vitro* (75). Finally, while this strategy has not been implemented in bacterial extract to our knowledge, translation factors may be depleted to create a platform to study and engineer their function.

#### Strain engineering to improve extract-based systems

Strain engineering is critical to create extracts which are optimized for high-level *in vitro* protein production. Genomic recoding, in which codons are systematically removed from the genome, is especially useful in engineering alterations to the genetic code in extract-based systems (76). The systematic global recoding of a codon to a synonymous alternative is required before its meaning can be changed without incurring detrimental or lethal effects. The power of recoding for *in vitro* ncAA incorporation was first demonstrated with the incorporation of the *p*-acetyl-l-phenylalanine (pAcF) at up to five sites in superfolder green fluorescent protein (sfGFP) in a partially recoded, release factor 1 (RF1) deficient strain, in which 13 occurrences of the amber stop codon (UAG) were reassigned to the synonymous UAA codon (77). Later, a fully recoded strain lacking all amber codons (C321.ΔA) with knockouts of RF1 and the phosphoserine (Sep) phosphatase SerB and introduction of a Sep orthogonal translation system (OTS) enabled site-specific incorporation of multiple Sep residues in a single protein in extract (78). This provided new insights into the role of serine phosphorylation on MEK1 kinase activity and increased the resolution at which phosphorylation-induced effects on protein structure and function can be defined, manipulated, and understood. Optimization of the fully recoded C321.ΔA – including the knockout of the genes *endA, gor, rne* and *mazF* – improved cell-free protein synthesis yields to >1.7 g/l in batch reactions and facilitated the incorporation of 40 identical pAcF residues site specifically into an elastin-like polypeptide with high ( ≥98%) accuracy of incorporation (10). More recently, Des Soye *et al.* modified the aforementioned optimized, fully recoded strain of *Escherichia coli* to express T7 RNA polymerase and enable high-yielding (∼2.7 g/l) cell-free transcription and translation reactions without exogenous polymerase addition (79). These yields outperform the best reported expression of proteins with single or multiple ncAAs *in vivo*. Overall, the ease of use and lower cost of extract-based protein synthesis makes it an attractive platform, and methods to gain greater control over reaction conditions are expanding the range of useful applications.

Taken together, the intensive development of both PURE and crude extract-based *in vitro* translation systems mean that an appropriate cell-free system is available for most engineering projects leveraging bacterial translation (nonbacterial cell-free translation systems are not covered in this review). However, when the goal is expansion or modification of the genetic code, the individual components of the translation machinery require special consideration. We next discuss the non-ribosomal components of the translation apparatus, what is known about their function, and how they may be engineered to enable *in vitro* alteration of the genetic code.

## tRNA ENGINEERING

Aminoacyl-tRNAs (aa-tRNAs) are at center stage during protein translation. They function as adapter molecules, enforcing the genetic code by recognizing the sequence information of an mRNA template and delivering their charged amino acid for incorporation into the growing polypeptide chain. More specifically, each tRNA must be (i) selectively charged by its cognate aminoacyl-tRNA synthetase (AARS), (ii) efficiently bound in its aminoacylated form by EF‐Tu for transport to the ribosome, and each must function optimally in translation by (iii) binding to the ribosomal A site, (iv) enabling peptidyl transfer, (v) translocating to the ribosomal P site, (vi) facilitating another acyl transfer reaction, and (vii) finally releasing from the ribosome. Below, we describe how tRNAs can be made, charged for use in *in vitro* reactions, and tuned for enhanced translation activity.

### *In vitro* transcription of tRNAs

Synthesis of tRNAs for cell-free protein synthesis can be done using *in vitro* transcription (IVT), wherein unmodified tRNA is synthesized by T3, T7 or SP6 bacteriophage systems (80). The T7 RNA polymerase (T7 RNAP) is most commonly used. T7 RNAP accepts linearized plasmid DNA, PCR products, or synthetic oligonucleotides templates containing a T7 promoter, and synthesis kits are commercially available. Importantly, the +1 nucleotide of the T7 promoter is usually a guanine or adenine and will be the template for the first nucleotide of the transcribed RNA. Moreover, two guanines following the +1 nucleotide greatly improve transcription yields (81). Due to these constraints, not every tRNA can be easily synthesized using T7 RNAP, and RNA yields vary with the sequence of the tRNA (82). To overcome this problem, self-excising ribozymes can be inserted between T7 RNAP promoter and tRNA template, enabling efficient transcription and control of the exact 5′ sequence (83,84). A similar trick can be applied to prevent T7 RNAP-mediated overextension at the 3′-end which would otherwise occlude the terminal adenine required for aminoacylation (84,85). Alternatively, DNA templates modified with methoxy moieties at the ribose C2′ position of the last two nucleotides help prevent non-templated nucleotide addition at the 3′ end (86,87). Although these tRNAs lack post-transcriptional modifications (88), a recent *tour de force* demonstrated that most of the 48 *E. coli* tRNAs can be synthesized using T7 RNAP and are functional in translation (74,82). Only five tRNAs (the isoacceptors for Glu, Asn and Ile) appear to require post-transcriptional modifications for activity.

### *In vitro* tRNA aminoacylation methods

**Figure 3.**
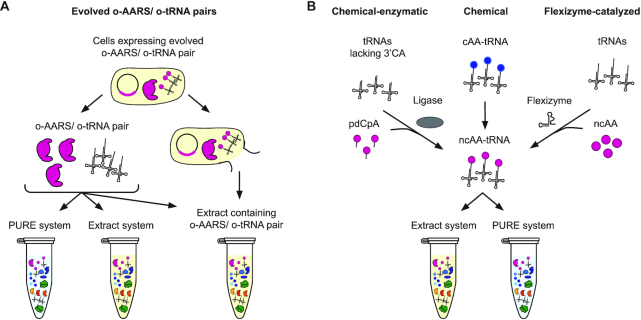
tRNA aminoacylation methods for non-canonical amino acid incorporation. Methods for tRNA aminoacylation may be divided into two categories – those which leverage engineered orthogonal variants of the protein aminoacyl-tRNA synthetases (*o*-AARS) used by organisms to charge tRNAs in cells, and those which bypass this system via alternative routes. Systems using an o-AARS/o-tRNA pair (left) follow one of two methodologies. In the first, the *o*-AARS and *o*-tRNA are individually purified and may be added at any desired concentration to either a PURE reaction or an extract-based reaction. If fewer purification steps are desired, the *o*-AARS/*o*-tRNA pair maybe expressed in cells from which an extract is directly prepared, alleviating the need to supplement it in the reaction, but ceding some control of reaction conditions. Alternative routes (right) are often used for monomers which do not have engineered o-AARS variants available. The first involves T4 ligase-mediated ligation of an aminoacylated pdCpA to a truncated tRNA (right-left). The second avoids the challenging ligation step by chemically modifying a monomer which is already aminoacylated to a tRNA by a native AARS (right-center). Lastly, artificial ribozymes called Flexizymes may be utilized to aminoacylate tRNAs with a wide range of non-canonical amino acids and other monomers (right-right). Once obtained and purified, these aminoacylated tRNAs may be used readily in a PURE or extract-based method for translation. Strain engineering or selective depletion can be used to modify the content of translational components in the extract.

To introduce ncAAs site-specifically into polypeptides, tRNAs need to be ‘misacylated’ with monomers beyond their native amino acids, which enables reassignment of codons to chemical substrates of interest. Four methods are used to generate ‘misacylated’ tRNAs *in vitro*: enzymatic aminoacylation via engineered AARSs, chemical-enzymatic aminoacylation, chemical modification of aminoacylated cognate amino acids, and flexizyme-catalyzed aminoacylation (Figure 3).

#### tRNA aminoacylation via engineered orthogonal AARS/tRNA pairs

In their native context, tRNAs are enzymatically aminoacylated with their cognate amino acids by highly specific AARS enzymes. These enzymes recognize their cognate tRNA substrates via identity elements in the tRNA’s acceptor stem, D-loop, variable loop, and the anticodon loop. The amino acid specificity is determined by the amino acid binding pocket and, in case of some AARSs, an additional editing domain which hydrolyzes misacylated tRNAs to ensure an accuracy in aminoacylation of at least 10 000:1 (89). Because of this stringent quality control, many ncAAs are not readily accepted by AARSs. To overcome this limitation, directed evolution has been used to generate orthogonal (*o*)-AARS/*o*-tRNA which charge the *o*-tRNA with desired ncAAs. In bacteria, the most widely used pairs are derived from the tyrosyl-(Tyr)RS/tRNA^Tyr^ pair from *Methanocaldococcus jannaschii* or the native amber suppressor pyrrolysyl-(Pyl)RS/tRNA^Pyl^ pair from *Methanosarcina barkeri*. Original innovations pioneered by Schultz and colleagues (90) have been adapted for many ncAAs, leading to, for example, *M. jannaschii* TyrRS/tRNA^Tyr^ pairs capable of installing diverse tyrosine derivatives (2), PylRS/tRNA^Pyl^ pairs incorporating lysine, phenylalanine and pyrrolysine derivatives, as well as click chemistry-reactive ncAAs (91), *S. cerevisiae* TrpRS/tRNA^Trp^ incorporating tryptophan derivatives (92,93), and *P. horikoshii* ProRS/tRNA^Pro^ allowing incorporation of proline derivatives (94). More recent work guided by crystal structures, genome engineering methods, and next generation sequencing, have pushed the limits of generating highly selective and orthogonal AARS/tRNA pairs that enhance the insertion of ncAAs into proteins. These include: compartmentalized partnered replication (CPR) (93), phage-assisted continuous evolution (PACE) (95), parallel positive selections (96), and multiplex automated genome engineering (MAGE) (97,98), among others. While many *o*-AARS/*o*-tRNAs pairs have been evolved *in vivo*, all of them can be used *in vitro* by adding them in purified form to either extract-based systems or the PURE system. In the case of extract-based systems, they also can be expressed in the extract source strain, circumventing time-consuming purification steps (99) (Figure 3A).

Despite many successful examples of changing the amino acid specificity of AARSs, these engineered enzymes still generally suffer from lower catalytic activity relative to their native counterparts. However, the use of these components *in vitro* provides a means to overcome catalytic inefficiencies, as the concentration of the *o*-AARS, the *o*-tRNA, and the ncAA can all be increased as required to attain robust ncAA incorporation. Accordingly, several groups have used extract-based systems with *o*-AARS/*o*-tRNA pairs to produce proteins containing ncAA via site-specific incorporation (10,77,100–105). In one example, Albayrak and Swartz demonstrated the potential of crude extracts to produce ncAA-containing proteins in higher yields by co-expressing an *o*-tRNA for amber stop codon suppression together with the target protein from one DNA template. In this way tRNA limitations could be overcome (105). While such innovations offer a work-around for low enzyme efficiencies, engineering orthogonal translation systems with high activity and specificity for a unique ncAA remains a significant systems-level challenge (106).

#### Chemical-enzymatic aminoacylation of tRNA

While AARS engineering is one approach to preparation of ‘misacylated’ tRNAs, this task can alternatively be accomplished using one of two chemical methods. The first approach combines chemical aminoacylation and enzymatic oligonucleotide ligation. This method involves synthesis and chemical aminoacylation of the hybrid di-nucleotide 5′-phospho-2′-deoxyribocytidylylriboadenosine (pdCpA) using an activated amino acid donor with an N-protected group followed by HPLC purification, concomitant ligation of the aminoacylated pdCpA to a truncated tRNA lacking the 3′terminal CA via T4 RNA ligase, and deprotection to liberate the free α-amino group (107–110) (Figure 3B, left). This approach is advantageous because it allows one to work with many different tRNAs to determine how the identity of the tRNA impacts the efficiency of *in vitro* translation. Additionally, a recent report suggests the potential for improved translation activity with artificial aminoacyl-tRNA substrates made with an *N*-nitroveratrylooxycarbonyl (N-NVOC)-monomer-pCpA synthesis method (111,112).

In the second method, canonical amino acids loaded onto tRNAs by their cognate AARS are subsequently chemically modified into ncAAs. For example, Fahnestock and Rich generated phenyllactyl-tRNAs through deamination of Phe-tRNAs with nitrous acid (15). Similarly, Merryman and Green generated tRNAs bearing *N*-monomethyl amino in a three-step process: protection of the α-amino group of the aa-tRNA using o-nitrobenzaldehyde, reductive methylation using formaldehyde, and deprotection by UV radiation to liberate the free α-*N*-methyl-amino group (14) (Figure 3B, center). Although these methods made ncAAs with bulky side chains accessible (such as glycosylated derivatives and larger organic fluorescent dyes (113,114)), and are applicable to nearly every ncAA in principle, they are technically demanding, laborious and often yield poor incorporation results due to the generation of a cyclic tRNA by-product which inhibits ribosomal peptide synthesis (115).

#### Flexizyme-catalyzed aminoacylation

Beyond protein-catalyzed and chemical charging approaches, ribozyme-catalyzed approaches also exist for acylating ncAAs to tRNAs. Specifically, small artificial ribozymes (44–46 nt) called Flexizymes (Fx) can be used to generate ncAA-tRNAs (116–118). Flexizymes originate from an acyl-transferase ribozyme (ATRib) capable of transferring N-biotin-Phe from the 3′-end of a short RNA to its own 5′-OH group (119). Through directed evolution and sequence optimization, ATRib was evolved into a family of three different Fxs (eFx, dFx and aFx) with different affinities to specific substrate-activating groups (120): eFx is used to acylate tRNAs with cyanomethyl ester (CME)-activated acids containing aryl functionality, dFx recognizes dinitrobenzyl ester (DNBE)-activated non-aryl acids, and aFx recognizes the hydrophilic activating group (2-aminoethyl)amidocarboxybenzyl thioester (ABT) which allows it to charge compounds with poor solubility in water.

Flexizymes selectively aminoacylate the 3′-OH of any tRNA, regardless of the body and anticodon sequences, with a broad range of carboxylic acids, including D- (18,121), β- (19), γ- (122) and other non-canonical amino acids (123) as well as N-alkylated amino acids (34,35) and even hydroxy acids (16,124), benzoic acids (125), exotic peptides (126) and foldamers (22) (Figure 3B, right). With the Flexizyme approach, a great variety of amino acids can be assigned to any tRNA as long as the amino acid side chain is stable during the esterification reaction and the monomer can be attached to the activated leaving group. Hence, in principle, the combination of Fx-catalyzed tRNA charging with an appropriate *in vitro* translation system allows near-total freedom in reassigning any codon with any ncAA. The most commonly used custom-made reconstituted translation system of this kind is called FIT (Flexible *In-vitro* Translation) system (118).

### tRNA engineering for improved ncAA incorporation

**Figure 4.**
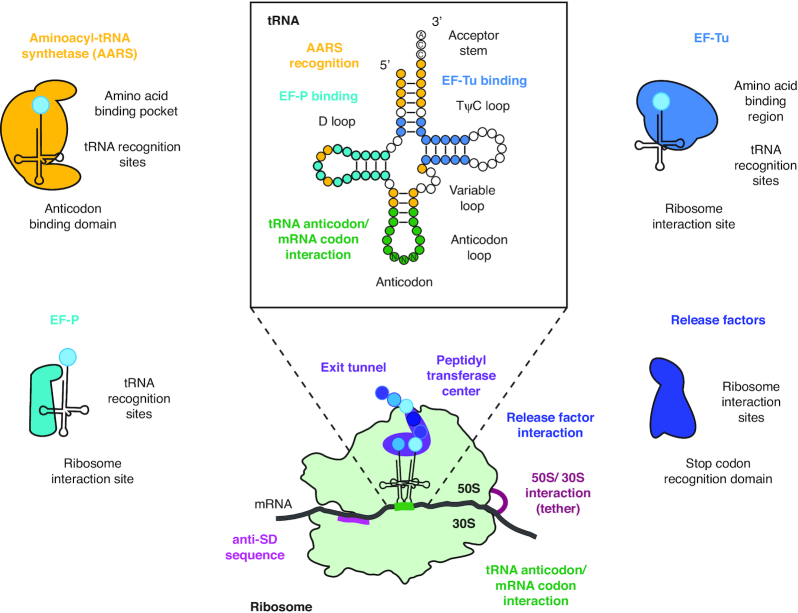
Engineering translation system components. tRNA (center-top) and ribosome (center-bottom) regions are labeled by name, and segments/ nucleotides that are known to be mediate specific processes of translation are labeled and color-coded by the translation factors they interact with. The most commonly engineered translation factors are depicted and labeled with regions of the molecule that may be targeted to modulate function.

The tuning of the translation machinery that has occurred through evolution has yielded tRNAs and translation factors—in particular Elongation Factor-Tu (EF-Tu)—with thermodynamic compensation interactions that are tuned to match canonical amino acids with cognate tRNAs. As a result, engineering aminoacyl-tRNAs with optimal loading properties for EF-Tu is important. To this end, key targets for tRNA engineering include mutations at or near the anticodon recognition domain of the AARS, as well as the T-stem region of the tRNA, which interacts with residues from the β-barrel domain 2 and the GTPase domain of EF-Tu (98) (Figure 4). In one example, Guo *et al.* evolved *M. jannaschii* tRNA^Tyr^_CUA_ variants for amber suppression by targeting regions implicated in EF-Tu binding (127). Modifications in EF-Tu binding regions of tRNA^Pyl^ also improved ncAA incorporation efficiencies using an o-PylRS/ o-tRNA^Pyl^ pair (128). The best variant of this study facilitated a 3-fold improvement in suppressing one amber codon and a 5-fold improvement when suppressing two. Complementary approaches involving engineering of EF-Tu itself have also been explored (discussed below).

## TRANSLATION FACTOR ENGINEERING

While the tRNAs are the adapters essential for decoding the mRNA message, various protein factors orchestrate the process of translation. They are responsible for initiating translation (IF1, IF2, IF3), choreographing translation through normal (EF-Tu, EF-G) or challenging (EF-P) sequences, and terminating translation at the three stop codons (RF1, RF2) (Figure 4). Considering the integral roles these factors play in the process, it is unsurprising that they are attractive targets for engineering translation *in vitro*. The following section surveys the roles of translation factors *in vitro*, summarizes work that has helped elucidate their functions, and describes their roles in promoting optimal translation in cell-free systems.

### Translation initiation engineering

In bacterial systems, translation is canonically initiated by the initiator tRNA (tRNA^fMet^_CAU_) which has been first charged with the initiator amino acid methionine (Met) and then formylated at the α-amino group on Met by methionyl-tRNA synthetase (MTF) to form fMet-tRNA^fMet^_CAU_. The fMet-tRNA^fMet^_CAU_ is then recruited by initiation factor 2 (IF2) to the 30S ribosomal subunit in the presence of all three initiation factors to form the 30S initiation complex (129). Considering this complex assembly of specialized initiator molecules, one might expect that engineering translation initiation would be a daunting task. However, pioneering works from the Schulman group demonstrated that protein synthesis can be initiated with non-methionine amino acids charged to tRNA^fMet^ with alternative anticodons. This implies that aspects of the tRNA^fMet^ (and not the attached amino acid or anticodon loop) are the primary selection determinant of IF2 for translation initiation (130). Later work using the PURE system demonstrated that 11 of the 19 amino acids other than Met were capable of initiating translation with greater than 50% the efficiency of wild-type initiation when charged to tRNA^fMet^_CAU._ A number of functionalized *N*^α^-acyl groups were also accepted, enabling spontaneous cyclization when paired with a C-terminal cysteine (131). D-amino acids acylated to tRNA^fMet^_CUA_ have also been demonstrated to competently initiate translation —especially when pre-acylated to mimic the formylated state—demonstrating that the chirality of the amino acid is not a requirement for translation initiation (121). The finding that formylation (or its mimic, acylation) improves but is not strictly required for translation initiation supports the hypothesis that the primary function of formylation is to discriminate against tRNA^fMet^_CAU_ binding to EF-Tu. This secures the role of tRNA^fMet^_CAU_ as solely an initiator tRNA by preventing sequestration by the highly abundant EF-Tu (132).

Beyond compatibility with non-canonical monomers, even short peptides acylated to tRNA^fMet^_CAU_ are capable of initiating translation – in some cases reported with even greater than wild-type efficiency (133). This technology was extended to enable the N-terminal incorporation of short peptide foldamers which were then cyclized using established techniques to generate peptides with defined and diverse structures (22,131). Notably, altering IF2 concentration did not improve foldamer incorporation in this study, despite the direct interaction between IF2 and tRNA^fMet^_CAU_ in the initiation process, suggesting that IF2 concentration is not limiting in 30S initiation complex formation. Supplementation of other initiation factors (IF1, IF3) or engineering of the ribosomal RNA itself may provide interesting targets for improving initiation with non-canonical substrates.

### Translation elongation engineering

After translation initiation, all the remaining amino acids in the production of a given polypeptide are added in the elongation phase. Thus, while translation initiation is critical, *in vitro* translation engineering must necessarily also focus on improving and modifying elongation, as this process is essential for the diversity of available monomers and the efficiency of polymer production. Such engineering efforts in elongation have significant barriers to overcome since, in contrast to initiation, elongation discriminates against some non-canonical monomers (e.g. D-amino acids).

With 10 or more copies present per ribosome, elongation factor EF-Tu is the most abundant protein in *E. coli* and is especially critical for efficient translation (134). EF-Tu is responsible for shuttling aminoacylated tRNAs to the ribosome while protecting against premature cleavage of the amino acid. The energetic interactions of each tRNA with EF-Tu combine with those of its cognate aminoacylated amino acid to produce a similar binding energy between each correctly aminoacylated tRNA and EF-Tu (135–137). This ‘goldilocks’ energy is strong enough to promote binding and protection of the aa-tRNA by EF-Tu, but weak enough to enable efficient release of the aa-tRNA for decoding in the A-site of the ribosome during elongation (138). The importance of this interaction for translation elongation has made EF-Tu an attractive target for modification to engineer non-canonical monomer incorporation – especially since monomers with large or negatively charged side chains are known to reduce binding affinity to EF-Tu (139,140). Foundational work demonstrated that the incorporation efficiency of ncAAs with bulky side chains and the preparation of tRNAs charged with ncAAs could be improved by utilizing an engineered version of EF-Tu with an enlarged binding pocket in the PURE system (141,142). In a series of similar efforts, randomization of the amino acid binding pocket of EF-Tu permitted the incorporation of the negatively-charged phosphoserine in vivo (143), later enabling milligram quantity production of phosphoproteins *in vitro* using cell extract from the recoded strain C321.ΔA (76,78), and increasing incorporation of *p*-azido-phenylalanine into proteins (104). A similar strategy was used to improve the EF-Tu guided incorporation of selenocysteine in the SECIS-free selenoprotein synthesis system (25). Later, the tRNA in this system was modified to encourage productive binding to EF-Tu and efficient decoding, as in the case of the engineered tRNAUTuX which was developed to improve selenocysteine incorporation and reduce serine misincorporation *in vitro* (144). Despite these key successes, engineering the binding pocket of EF-Tu is not always an effective strategy, and was detrimental to the incorporation of D-amino acids in one study (145). Future projects in this space may benefit from the throughput and ability to test many combinations of variants provided by *in vitro* systems (104).

During the elongation process, peptide bond formation efficiencies depend on the steric and reactive properties of the AA-tRNAs in the ribosomal active site. Hence, incorporation of several ncAAs with non-canonical backbones such as D-α- and β-amino acids have suffered from low efficiencies. EF-P is a bacterial translation factor that accelerates peptide bond formation between consecutive prolines (146,147). It has been shown that the requirement of EF-P stems from the imino acid's low reactivity, steric orientation, and rigidity in the ribosomal active site, and not from a requirement of tRNA^Pro^ (148). Furthermore, it has also been shown that the identity element for EF-P binding is the 9-nt D-loop found in tRNA^Pro^ isoacceptors, and not proline itself (149) (Figure 4). EF-P then binds to the P-site peptidyl-Pro-tRNA^Pro^ to promote peptide bond formation with the A-site Pro-tRNA^Pro^, preventing ribosome stalling and accompanied peptidyl-tRNA drop-off. Given the non-specificity for the charged amino acid and its ability to facilitate translation of challenging sequences, the potential of EF-P to facilitate translation engineering is compelling.

The complex interplay between the elongation factors, tRNAs, and the ribosome highlight the need for multi-component engineering of the translation system. The flexibility of cell-free systems may offer some advantages here. For example, Katoh *et al.* generated a hybrid tRNA, called tRNA^Pro1E2^, consisting of the T-stem motif of *E. coli* tRNA^Glu^ as well as the D-arm motif of *E. coli* tRNA^Pro1^ to facilitate improved synthesis efficiencies based on tighter binding to EF-Tu. The T-stem motif derived from tRNA^Glu^ has a high binding affinity to EF-Tu, so by combining this motif with the D-arm motif of tRNA^Pro1^, the authors compensated for the general low affinity of D-α- and β-aminoacyl-tRNAs toward EF-Tu, improving peptide bond formation. This enabled enhanced incorporation of D-amino acids and consecutive incorporation of β-amino acids, especially when adding EF-P to the translation system (20,24). Combining these efforts with engineering of EF-Tu may further improve this system (141–143).

### Translation termination engineering

Translation is terminated at the codons UAA, UAG and UGA by the release factors RF1 (UAA, UAG) and RF2 (UAA, UGA). Since genetic code expansion efforts have traditionally targeted the rare UAG stop codon for recoding, the deletion of RF1 was an important goal to minimize errant truncation at UAG codons intended for recoding. This goal was first achieved by partial removal (150,151), and later, as described above, complete removal (76) of the UAG codon from the *E. coli* genome, which permitted the genomic deletion of RF1, enabling complete reassignment of the amber codon translation function in strain C321.*ΔA*.

While genome-wide substitution of stop codons by defined synonyms recoding has made a tremendous impact on efforts to site-specifically incorporate ncAAs into proteins, engineering of release factors themselves has so far been limited. Toward the goal of making an ‘omnipotent’ release factor which terminates at all three stop codons, modification of RF2 at position 213 with the corresponding RF1 residue reduced its discrimination against termination at the UAG stop codon, though with greatly reduced overall termination efficiency in *in vitro* competitive peptide release experiments (152). In the opposing direction, one could imagine reducing the number of viable stop codons targeted by an orthogonal release factor to open multiple stop codons for recoding *in vitro*, or even engineering the RF to target a sense codon.

## ENGINEERING THE RIBOSOME

As the central catalytic machine facilitating peptide bond formation, the ribosome is an obvious target for engineering translation. Though it has evolved to build polypeptides out of the canonical set of 20 amino acids, the wild-type ribosome is also capable of producing polymers with non-peptide backbones (e.g. polyesters), and has even incorporated select exotic monomers and foldamers (153). Although many types of monomers can be incorporated into a growing polymer chain by the ribosome, incorporation of backbone-modified monomers (e.g. cyclic, β- or D-amino acids) remains limited in the total peptide length and incorporation efficiency due to reduced compatibility with the ribosome's catalytic active site and nascent peptide exit tunnel (154–156). As described above, other facets of the translation system (tRNAs, EF-Tu, etc.) must be tuned to facilitate the use of such monomers. Below, we discuss recent advances that set the stage to engineer the ribosome itself, which include methods for purification, reconstitution, synthesis, engineering, and evolution of the ribosome *in vitro*.

### Ribosome reconstitution

**Figure 5.**
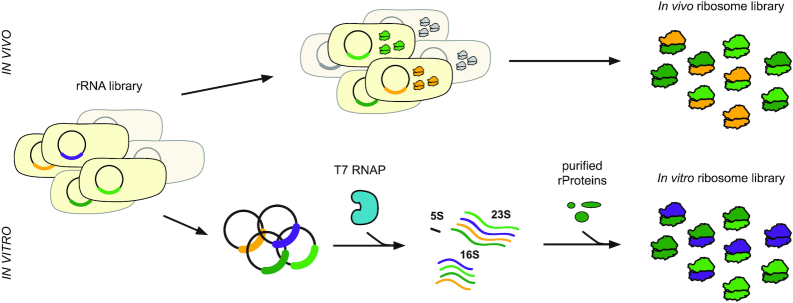
Generation of ribosomal variants for engineering the translation machinery *in vitro*. Ribosome libraries can be generated by two main strategies. In one approach, ribosome libraries are built *in vivo* and entail transformation of a library of rRNA variants, expression of those variants in living cells, and purification of fully assembled ribosomes for *in vitro* manipulation (top). One drawback of this method is that dominant lethal genotypes—those which kill any cell in which they are expressed—will not be present in the final library (grayed out cells). In contrast, methods for building ribosomes purely *in vitro* can avoid this constraint, enabling the construction of many ribosomal variants which may be lethal *in vivo* (purple). It is possible, however, that this library may be missing ribosomal variants which have difficulty assembling properly *in vitro* (yellow).

The ribosome is composed of a small (30S) and large (50S) subunit. The 30S subunit decodes mRNA and accommodates corresponding tRNA-monomers. The 50S subunit accommodates tRNA-monomers, catalyzes polypeptide synthesis, and excretes polypeptides. Structure, function and assembly studies of the ribosome have transformed our understanding of the ribosome's parts, defined functional relevance, and elucidated mechanism. This has been greatly facilitated by ribosome reconstitution and purification approaches. Traditional methods for isolating ribosomes for testing *in vitro*, including mutant versions, involve purification via ultracentrifugation and supplementation of *in vivo*-synthesized ribosomes or ribosomal RNA (rRNA) into expression reactions (157). While this methodology allows a great deal of study into ribosome structure and function, it has been limited in its ability to generate some mutant rRNAs, which result in lethality in the cells which depend on them for protein synthesis (Figure 5, top). To circumvent this limitation, several groups have developed strategies for plasmid expression and purification of affinity-tagged rRNA variants to circumvent this difficulty, enabling *in vitro* characterization of otherwise inaccessible mutants (158–161). Similar methods were later used to isolate ribosomes evolved for orthogonal templates and enhanced genetic code expansion for testing *in vitro* (162). These ribosomes may be directly supplemented into *in vitro* translation reactions for dissection of ribosome function, or the rRNA and protein components can be isolated and recombined in ribosome reconstitution experiments to study ribosomal subunit assembly.

#### 30S subunit reconstitution

As an alternative to building ribosomes in cells, followed by their purification, ribosomes can be assembled, or reconstituted from *in vivo* purified components or *in vitro* synthesized parts. In a ground-breaking paper, Taub and Nomura demonstrated for the first time that isolated 16S rRNA and the total complement of 30S ribosomal proteins (TP30) were sufficient for reconstitution of functional *E. coli* 30S particles in a single step (163). This simple strategy, when coupled with advancing imaging techniques, was enough for the elucidation of much of the 30S assembly process (164–166). Initial assessments of relationships between ribosomal proteins in the structure of the ribosome were determined via chemical iodination studies (167). It was later shown that 30S particles could be assembled from TP30 and *in vitro* synthesized 16S rRNA lacking base modifications, demonstrating that these modifications are not required for 30S assembly. These particles formed 70S ribosomes when supplied with purified 50S particles which were capable of A- and P-site binding, but not peptidyl transferase activity (168). Ribosome biogenesis factors play an important role in the assembly of functional ribosomes *in vivo* and have been shown to facilitate 30S assembly *in vitro* (169). Leveraging this knowledge, Tamaru *et al.* were able to synthesize 30S particles from 16S rRNA and 30S r-proteins all individually purified from cells and assembled in the presence of ribosome biogenesis factors and under physiological conditions (170). Assembly of 30S particles from independently generated components can provide exquisite control over assembly and function of the translation machinery, facilitating engineering applications. Li *et al.* achieved this milestone by synthesizing 30S particles from fully *in vitro* generated 16S rRNA and TP30 supplemented 30S ribosome assembly factors to assemble 30S particles with 21% activity of native ribosomes (171). Taken together, recent advances in 30S reconstitution have begun the march towards building ribosomes *de novo* in test tubes, as well as mutant ribosomes capable of enhanced synthesis of proteins with ncAAs.

#### 50S subunit reconstitution

While significant strides have been made in bottom-up synthesis of 30S subunits, *in vitro* generation of active 50S particles has posed a greater challenge. Reconstitution of the 50S ribosomal subunit was first achieved by Nierhaus and Dohme, but required a two-step incubation at high salt concentration and temperature (172). Despite the non-physiological nature of this reconstitution, this methodology combined with scanning transmission electron microscopy was instrumental in elucidating both the order and dependencies of r-protein binding and 50S assembly (173). However, 50S subunits reconstituted in this way are less active than those purified from cells (174). Single-step reconstitution of large subunits from the halophilic *Haloferax mediterranei* has been achieved in the presence of high (2.5 M) monovalent cation and 60 mM magnesium concentrations at the optimal growth temperature (40–45°C) for this organism, indicating that this two-step requirement is not universal across diverse organisms (175).

In generating mutant 50S particles, it is desirable to use *in vitro* transcribed 23S rRNA, whereby mutations can be easily introduced without cell-viability concerns. *In vitro* transcription and methylation studies of the *E. coli* 23S rRNA from a linearized template using T7 RNA polymerase were the first step toward synthetically produced large ribosomal subunits (176). Unfortunately, rRNA produced in this way does not assemble correctly into *E. coli* 50S particles – a deficit which was attributed to a lack of critical base modifications to the synthetic transcript which are present in wild-type 23S (174). However, *in vitro* transcribed rRNA from two thermophiles were able to be assembled into functional large subunits in a one- or two-step treatment with high-salt, indicating that these modifications are not in fact essential for ribosome function (177,178). Later experiments demonstrated that the addition of osmolytes to *in vitro* transcribed reconstitution reactions of *E. coli* 23S rRNA resulted in a 100-fold improvement in the assembly of active large subunits (179). However, 50S particle reconstitution still relies on 50S proteins purified from cells – assembly of 50S particles from fully *in vitro* synthesized parts as was achieved with the 30S in (171) has not yet been reported.

### Integrated ribosome synthesis and assembly

The ability to reconstitute both the small and the large subunit of the ribosome from *in vitro* transcribed RNA suggested that a fully integrated approach to ribosome assembly might be possible. In developing such a method, it was important to identify conditions which would promote the assembly of both subunits under similar, ideally physiological, conditions. Carrying out transcription of rRNA in a cell extract which mimicked the cytoplasm (180) was identified as a promising strategy to enable assembly of both subunits under similar, physiological conditions. Indeed, by expressing the entire *rrnB* operon under the control of the T7 promoter in ribosome-depleted cell extract supplemented with TP70, integrated synthesis, assembly, and translation (iSAT) of a reporter protein was demonstrated in a single reaction (75). This method enables synthesis and testing of ribosomes that would be dominant lethal or otherwise inviable *in vivo* (Figure 5, bottom). Further optimization of the genetic construct encoding the ribosomal operon yielded a 45-fold improvement in ribosome activity in iSAT (181) and crowding and reducing agents conferred a further ∼4-fold improvement (182). Carrying out iSAT in a semi-continuous reaction vessel to allow diffusion of waste products and energy sources yielded another 7-fold improvement in protein yields (183). An evolutionary design of experiment approach was able to identify further optimizations to reduce reagent use as well as implement a less costly energy source – an approach which holds promise for further optimizations of the platform (31).

### Ribosome evolution and engineering

While capabilities are emerging to build ribosomes from the ground up, parallel efforts have reached important milestones for engineering extant ribosomes. Key among these is atomic mutagenesis, an elegant technique pioneered by the Polacek lab which uses *in vitro* assembly of 50S particles to introduce atomic mutations and other non-canonical modifications into 23S rRNA (reviewed in (184)). Leveraging classical ribosome complementation assays (174) and employing chemically synthesized RNA oligonucleotides as the complementing fragment, researchers may incorporate bases with atomic level modifications to dissect the role of individual nucleobases in various aspects of ribosome function (185). This method has been used to elucidate the role of specific nucleotides in peptide bond formation (186), EF-G triggered GTP hydrolysis (187,188), and tRNA binding (189). Atomic mutagenesis of mRNA has also been utilized to elucidate the mechanism of stop codon recognition by release factors (190). Looking forward, this approach appears to be promising for constructing a new generation of engineered ribosomes whose chemical activity and substrate specificity can be augmented by adding artificial nucleotides into 23S rRNA.

Towards evolution of the ribosome for new functions, several projects to date have pursued ribosome directed evolution using *in vivo* systems, and have enjoyed noteworthy successes such as ribosomes which are orthogonal (191) and release factor resistant (192), or capable of quadruplet decoding (193), or β-amino acid incorporation (194,195) among others. Unfortunately, such efforts are constrained by the fact that cell-viability limits the changes that can be made to the ribosome, with many mutations conferring dominant lethal phenotypes. Recently, Orelle *et al.* addressed this gap through the construction of the first complete functionally orthogonal ribosome-mRNA system in cells, where a sub-population of ribosomes are available for engineering and are independent from wild-type ribosomes supporting cell life (196). This was achieved by constructing a ribosome with covalently tethered subunits called Ribo-T (the core 16S and 23S ribosomal RNAs form a single chimeric molecule with the connection at where helix h44 of the 16S rRNA and helix H101 of the 23S rRNA). Ribo-T was evolved by selecting otherwise dominantly lethal rRNA mutations in the PTC that facilitate the translation of problematic protein sequences not accessible to natural ribosomes. Similar tethered, or stapled ribosomes, were used to access new ribosome function (197,198), as well as incorporate ncAAs. Unfortunately, covalently linked ribosomes appear to have reduced rates of ribosome assembly, initiation, and termination (199). Despite this, they serve as a key first step to being able to engineer the large subunit of the ribosome towards the polymerization of monomers with non-canonical backbones (196,198,200).

Curiously, despite the development of an *in vitro* ribosome selection strategy 15 years ago (29), little ribosome evolution work has been done *in vitro* since that time. Perhaps this is because the ribosomes selected in this study were still produced from an *in vivo* produced library, and therefore performing the selection *in vitro* represents an unnecessary added complexity. However, there are advantages to performing selections *in vitro*, including a greater degree of control over selection conditions, the potential for larger library sizes, and enhanced throughput. The development of the ability to synthesize functional ribosomes *in vitro* in the iSAT system may make *in vitro* selection of the ribosome more attractive, and forthcoming work will set the stage for the development of this technology. To advance this mission, *in vitro* encapsulation may prove useful (201,202).

## CONCLUSIONS AND FUTURE DIRECTIONS

Looking forward, we anticipate that continued developments of *in vitro* translation system engineering will have fundamental importance and significant practical applications. First, new research will help shed light on the intricacies of bacterial translation. Translation is a highly complex system, and just as successful examples of engineering the translation apparatus illustrate our understanding of it, instructive failures reveal gaps in our current understanding. By engineering bacterial translation to accommodate novel monomers, we will dramatically perturb each step of translation and allow new biochemical analysis of individual steps. This fundamental knowledge has been previously difficult to obtain with available wild-type translation components and traditional mutagenesis strategies, and is still constrained in cells by viability concerns.

Beyond fundamental scientific breakthroughs in the biology of translation, new advances could also inform our understanding of life's origins. According to the RNA World hypothesis, protein translation, especially the origin and evolution of the ribosome, shepherded the transition from the primordial biota relying on RNA to the modern world (203). The high conservation of the catalytic core of the ribosome raises fundamental questions about the origin and evolution of the translation system (204,205). Understanding the structural and substrate flexibility of the ribosome may provide important experimental insights that are currently missing.

With respect to emerging opportunities, the ability to produce peptides and polymers comprised of only non-proteinogenic monomers could lead to new applications based on novel sequence-defined polymers, such as new classes of peptidomimetic drugs or advanced materials. Just as organic chemistry once revolutionized the ability of chemists to build molecules following a basic set of design rules, so too will new foundational technologies for the biosynthesis of sequence defined polymers from engineered translation machinery provide a transformative toolbox for synthetic and chemical biology.
